# Barriers to monitoring and management of cardiovascular and metabolic health of patients prescribed antipsychotic drugs: a systematic review

**DOI:** 10.1186/s12888-020-02990-6

**Published:** 2020-12-04

**Authors:** Ruba Azfr Ali, Zahraa Jalal, Vibhu Paudyal

**Affiliations:** 1 School of Pharmacy, University of Birmingham, Birmingham, UK; 2 Clinical Pharmacy Department, Umm Al-Qura University, Makkah, Kingdom of Saudi Arabia

**Keywords:** Antipsychotics, Comorbidity, Drug interactions and side effects, Patient Counselling research, Primary care

## Abstract

**Background:**

The use of atypical antipsychotics which currently form the primary choice pharmacotherapy for several mental health conditions have been linked to cardiovascular and metabolic side effects. This systematic review aimed to investigate the barriers to monitoring and management of cardiovascular co-morbidities in patients prescribed antipsychotic medicines.

**Methods:**

A protocol-led (CRD-42018106002) systematic literature review was conducted by searching Medline, Embase, and PsycINFO databases 2003 until October 2019. Cochrane, Centre for Review and Dissemination (CRD) and PRISMA guidelines were followed. Studies investigating barriers to monitoring and management of cardiovascular co-morbidities in patients prescribed antipsychotic medicines were included.

**Results:**

A total of 23 records were included. Key barriers included a) health-care system-related factors such as lack of knowledge and expertise amongst care providers, available resources, confusion around remit and roles, fragmentation of care such as across general practitioners and psychiatrists, and time constraints and b) patient-related factors such as disability resulting from mental health conditions, knowledge and skills of the patients.

**Conclusion:**

Barriers to monitoring and management of cardiovascular and metabolic health of patients taking antipsychotic medicines are multidimensional. Apart from educational interventions directed to both patients and health-care professionals, the results suggest a need for the improvement of wider system-related factors to improve physical health of patients prescribed antipsychotic medicines.

Clearer guidelines, clarity of remit and roles amongst service providers are necessary in addition to educational interventions directed at patients and health-care professionals in improving physical health monitoring, counselling and management of patients prescribed antipsychotic medicines.

**Trial registration:**

A protocol was developed and registered with PROSPERO as per PRISMA-P guidelines (CRD 42018106002).

**Supplementary Information:**

The online version contains supplementary material available at 10.1186/s12888-020-02990-6.

## Background

Patients with severe mental illnesses face inequality in health and access to healthcare and face early deaths. For example, a recent meta-analysis suggested that schizophrenia is associated with a weighted average of 14·5 years of potential life lost, with females being particularly disadvantaged over males [1]. Cardiovascular disorders including coronary heart disease and cerebrovascular disorders disease are the leading cause of deaths in persons with severe mental illnesses [1, 2].

Atypical antipsychotics which currently form the primary choice pharmacotherapy for the management of psychotic episodes in schizophrenia, bipolar disorders and other several mental illnesses, have been linked to cardiovascular and metabolic side effects [1]. Patients with severe mental illnesses such as schizophrenia are at dual disadvantage of being inherently predisposed to metabolic abnormalities and often this is worsened by the subsequent use of antipsychotics [1]. Results from a meta-analysis assessing the prevalence of metabolic abnormalities between antipsychotic naïve patients and chronically treated found that the rate of individual metabolic abnormalities was significantly higher in the chronically treated group [3].

In 2003, the Food and Drug Administration (FDA) launched a black box warning regarding the diabetogenic effect of antipsychotics [4]. In response to these warnings, a panel of regulatory bodies and professional associations including the American Diabetes Association (ADA)/American Psychiatric Association (APA) published a consensus statement that recommends early and regular monitoring of metabolic side effects among antipsychotics users [5].

Recently the National Institute for Health and Care Excellence (NICE) guidelines for managing schizophrenia have introduced updates to include management of cardiovascular and metabolic abnormalities in patients with schizophrenia [6]. The updates included: measuring bodyweight (plotted on a chart); waist circumference, pulse and blood pressure, fasting blood glucose, glycosylated haemoglobin (HbA1c), blood lipid profile and prolactin levels. Additional measures including electrocardiogram (ECG) should be conducted for patients with specific cardiovascular risk such as diagnosis of high blood pressure, medical history of cardiovascular disease and during hospital admission.

Despite current guidelines, rate of screening and management of metabolic and cardiovascular health has been reported to be sparse. A study exploring the prevalence of undiagnosed metabolic abnormalities in patients with severe mental illnesses in England, reported a very low proportion of patients had undergone annual screening and monitoring for cardiovascular risks factors such as blood pressure (2%), weight (0%), waist circumference (0%), any glucose (7%) and lipid profile (4%) [7].

Despite the positive outcomes which have been associated with the introduction of atypical antipsychotics on the quality of life of people with severe mental illness, [8] the associated cardiovascular and metabolic risks of these drugs may limit their use. Given that cardiovascular disorders are leading cause of deaths in patients with severe mental disorders and quantitative data suggesting suboptimal follow up and monitoring practices, it is important to understand the factors associated with sub-optimal monitoring, counselling and management of cardiovascular and metabolic side effects that may be contributing to excess morbidity and mortality in this population.

This study aimed to systematically review the barriers to the monitoring, counselling and management of cardiovascular and metabolic side effects in patients taking antipsychotic medicines.

## Methods

### Search strategy and eligibility criteria

A protocol was developed and registered (CRD 42018106002). Electronic search was performed for literature from 2003 until October 2019 using Medline, Embase, and PsycINFO databases. Search was limited to English language due to constraints in time and funding to undertake the translation. This time frame was selected as it corresponds to the FDA warnings for risks of metabolic dysregulations associated with antipsychotic medicines. A search strategy was developed based on keywords and medical subject headings (Electronic supplementary material 1). Cochrane, Centre for Review and Dissemination (CRD) and PRISMA guidelines (Electronic supplementary material 2) were followed in conducting and reporting the review.

The inclusion criteria were formulated using the PIC/o mnemonic for qualitative evidence which stands for participants, phenomenon of interest and the context [9]. Key inclusion criteria were: (i) patients aged ≥18 years old using one or more antipsychotics for severe mental illnesses; (ii) health care professionals who are involved in the care of the patients which reported barriers to screening, monitoring and management of cardiovascular and metabolic side effects in patients prescribed antipsychotic medicines. Studies which did not specify patient antipsychotic use as inclusion criteria, but reported participant perspectives around barriers to monitoring of cardiovascular or metabolic health in the context of the drug use were also included in the review.

### Study identification and data extraction

Title and abstracts were screened for eligibility. After duplicates removal, records with irrelevant titles or abstracts were excluded from the search. Two reviewers undertook the screening and reviewing process independently. Conflicts regarding the results were resolved by consensus or after a discussion with a third reviewer.

The extracted data included: participants characteristics, data collection method, phenomenon under the study and the study’ main findings. Each finding was assigned a level of credibility: Unequivocal [U], Credible [C], or Unsupported [US], according to the criteria from the Joanna Briggs Institute [10]. Findings were rated as unequivocal if they were directly supported by illustrations. Findings were labelled as credible if they were indirectly cited in the original study (derived from the meaning). If the findings were drawn from the authors’ conclusion, the reviewer would label them as unsupported. Inductive thematic synthesis approach was applied. Quality assessment was done using Joanna Briggs Institute Assessment and Review Instrument (JBI-QARI) for qualitative studies.

## Results

### Included studies

**Fig. 1:**
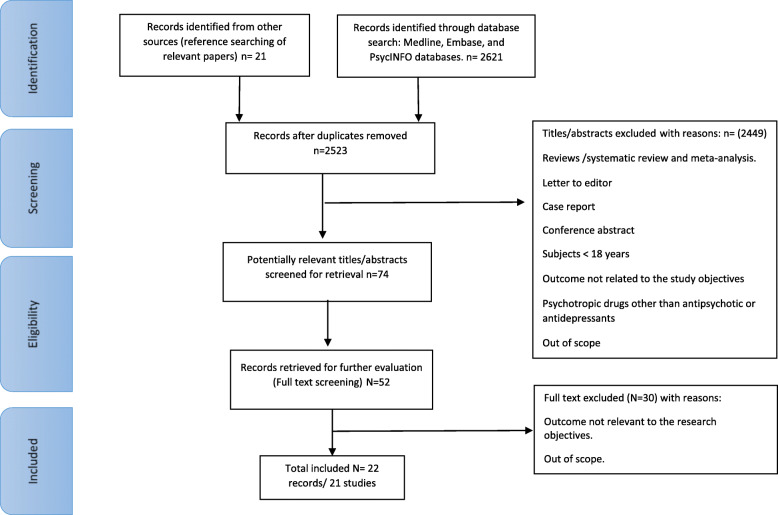
PRISMA flowchart of the selection process

A total of 22 records were included in the final synthesis which related to 21 studies [11–31]. (Fig. 1).

### Study characteristics

The majority of the studies were conducted in Europe [11, 13–15, 17, 19, 21, 23, 26, 29] and America [12, 16, 22, 24, 25, 27, 30]. Five studies recruited participants from community mental health settings, [18, 21, 26, 29, 30] whereas 10 studies recruited participants from secondary care settings [12, 14–17, 19, 22, 24, 27, 31] and two recruited participants from tertiary centres [11, 23] Four studies did not specify the setting type [13, 20, 21, 25, 28]. A total of 10 studies investigated patients’ views [12, 13, 15–18, 24, 27, 29, 30] and 10 studies focused on providers’ prospective [11, 19–23, 25, 28, 31]. Only two studies, [14, 26] examined the perspective of both mental healthcare professionals and patients.

Among the studies that included patients, only three studies exclusively targeted regular antipsychotic drug users [17, 24, 29]. The majority of the patient focused studies recruited participants based on clinical diagnoses with mental health conditions but included patient perspectives in relation to cardiovascular and metabolic health monitoring and hence were included [13–16, 18, 25–27, 30]. Five of these studies focused on participants diagnosed with psychosis [14, 15, 17, 24, 29] while four studies included wide range of mental health conditions [12, 16, 18, 26].

Various range of healthcare professionals were included in the studies that looked at healthcare professionals’ perspectives, [11, 14, 19–23, 25, 26, 28, 31] including mental health nurses, [11, 20, 23] community mental health staffs [20, 21, 31] and primary care providers [22].

Full study characteristics are reported in Tables 1 and 2.

**Table 1:** Characteristics of studies including patients

Study ID & Country	Aim	Design	Data collection method	Setting	Sample size	Participants	Mental health diagnosis	Proportion of patients prescribed antipsychotic drugs	Information on the use of antipsychotic by patients
Johnstone, 2009;UK [29]	To investigate the barriers to uptake of and adherence to physical activity in community dwelling patients with a diagnosis of schizophrenia	Qualitative study	Semi-structured interviews	Community mental health services	27 community dwelling patients with a diagnosis of schizophrenia	Patients with mental illness	Patients with diagnosis of schizophrenia for a minimum of 1 year	100%	Second generation antipsychotic 52% Clozapine 44% First generation antipsychotic 4%
Rastad, 2014; Sweden [17]	To study the perception and experience of barriers to and incentives for physical activity in daily living in patients with schizophrenia, as reported by the patients themselves.	Qualitative exploratory study	Semi-structured interviews	Multicentre (3 psychiatric outpatient clinics)	20 patients with schizophrenia or schizoactive disorder	Patients with mental health conditions	Patients with schizophrenia or schizoactive disorder	100%	No data available on the type of antipsychotics
Vandyk, 2012; Canada [24]	To explore the subjective experience of weight and lifestyle from the perspective of people with schizophrenia	Qualitative study within constructivism paradigm	Interview	Outpatient clinic at psychotic hospital	19 patients diagnosed with schizophrenia or schizoactive disorder	Patients with mental health conditions	Schizophrenia or schizoactive disorders	100%	Atypical antipsychotics100%, conventional antipsychotics 39%
Blixen, 2016;USA [12]	To assess perceived barriers to self-management among patients with both SMI and DM in order to inform healthcare delivery practices	Exploratory, observational and phenomenological approach	Interview: semi-structured	Urban safety net clinic	20 subjects with severe mental conditions	Patients with severe mental illness	schizophrenia, schizoactive disorder, bipolar disorder or major depression	Not reported	No specific information regarding the type of antipsychotic drug use however participants described barriers to monitoring and management of cardiovascular and metabolic side effects
Graham, 2014; Canada [16]	To potentially enhance health initiatives by exploring facilitators that help mental health service users engage in better health behaviours and the types of health programs mental health service users want to develop	Qualitative study	Focus group interviews (5 groups)	Psychosocial rehabilitation centre	37 mental health service users	Patients with mental health conditions	Broad range of mental conditions (Schizophrenia, bipolar related disorders, depressive disorders, anxiety disorders, obsessive-compulsive disorders)	Not reported	No specific information regarding the type of antipsychotic drug use however participants described barriers to monitoring and management of cardiovascular and metabolic side effects
Kristiansen,2015; Denmark [14]	To explore physical health problems and their causes in patients with severe mental illness, as well as possibilities for prevention and treatment from the patients’ and staff’s perspectives	Qualitative study	Focus group interviews (6 groups)	3 outpatients clinics	33 participants: staff: *n* = 19 and patients: *n* = 14.	Mental health care staff and patients with mental health conditions	Patients with schizophrenia or substance abuse	Not reported	No specific information regarding the type of antipsychotic drug use however participants described barriers to monitoring and management of cardiovascular and metabolic side effects
McDevitt, 2006;USA [30]	To explore perceived barriers and benefits to physical activity in people with serious and persistent mental illness (SPMI) who were enrolled in community-based psychiatric rehabilitation.	Qualitative exploratory study	Focus group interviews (4 groups)	2 community based psychiatric rehabilitation centres	34 participants	Patients with mental health conditions undergoing community based psychiatric rehabilitation	No specific information regarding the diagnosis of mental conditions	Not reported	No specific information regarding the type of antipsychotic drug use however participants described barriers to monitoring and management of cardiovascular and metabolic side effects
Pearsal, 2014; UK [18]	To examine the attitudes, views, and experiences of patients who had declined to participate in a healthy living programme	A qualitative study using grounded theory	Interview	Community mental health services	30 patients with a diagnosis of schizophrenia, schizoaffective or bipolar affective disorder	Patients with mental health conditions declined to take part in a healthy living programme based in a community mental health team	Patients with schizophrenia, schizoaffective or bipolar affective disorder	Not reported	No specific information regarding the type of antipsychotic drug use however participants described barriers to monitoring and management of cardiovascular and metabolic side effects
Pitman, 2011; UK [26]	To assess current practice and attitudes related to screening and reduction in risk factors for cardiovascular disease, preferences for service provision, and perceived barriers to service uptake.	Cross sectional study	Survey	Community psychiatric services	227 service users, 143 primary care staff, and 166 community mental health team (CMHT) Staff.	Mental health care staff and patients with mental health conditions	Patients with wide range of mental conditions (Schizophrenia, bipolar disorder, depression and anxiety)	Not reported	No specific information regarding the type of antipsychotic drug use however participants described barriers to monitoring and management of cardiovascular and metabolic side effects
Yarboroug,2011;USA [27]	To adapt a successful lifestyle/weight loss intervention for this population, deliver it in mental health clinics, and concurrently measure implementation factors	Mixed methods: Qualitative interviews with intervention observation sessions	Interview	Two public mental health clinics	Control group (*N* = 32) Intervention group (*N* = 16)	Patients with mental health conditions	No specific information regarding the diagnosis of mental conditions	Not reported	Report mentions that patients should be stable on antipsychotic therapy ≥30 days, but no specific information regarding type of antipsychotics; Participants described barriers to monitoring and management of cardiovascular and metabolic side effects
Yarboroug, 2016; Portland [13]	To identify modifiable factors associated with making and maintaining healthy lifestyle changes in order to inform clinicians and improve the development of future interventions for individuals with serious mental illnesses	Mixed methods: Qualitative interviews with intervention observation sessions	Interview	Multicentre (unclear)	84 participants	Patients with mental health conditions	No specific information regarding the diagnosis of mental conditions	Not reported	Report mentions that all patients should be stable on antipsychotic therapy ≥30 days, but no specific information regarding type of antipsychotics; participants described barriers to monitoring and management of cardiovascular and metabolic side effects
Wärdig, 2015; Sweden [15]	To describe how persons with psychosis perceive participation in a lifestyle intervention, and use these perceptions to present factors to for consideration in future interventions	Qualitative, phenomenological approach	Interview: semi-structured	Psychiatric outpatient care	40 participants with psychosis	Patients with severe mental illness	Patients diagnosed with psychosis	Not reported	The participants were on outpatient care with diagnosed psychosis and medicines use

**Table 2:** Characteristics of studies not involving patients as participants

Study ID & Country	Aim	Design	Data collection method	Setting	Sample size	Participants	Information on the use of antipsychotic by patients
Bergqvist, 2013; Sweden [19]	To explore mental health professionals’ experiences of assisting people with a psychotic disorder to implement lifestyle changes in an effort to prevent metabolic syndrome.	Phenomenology	Interview	Psychiatric outpatient care.	12 health care staff members (9 specialised nurses, 2 Outpatients’ therapists, 1 nursing assistant.	Mental health care staffs	Participants described barriers to monitoring and management of cardiovascular and metabolic side effects
Happell, 2013; Australia [20]	The current study seeks to explore nurse views on screening/monitoring of the physical health of consumers with SMI, in mental health care.	Qualitative exploratory study	Focus group interviewers (6 groups)	Regional mental health care service	38 nurses	Community mental health nurses	Nurses described barriers to monitoring and management of cardiovascular and metabolic side effects amongst their patients
Hultsjö, 2012; Sweden [21]	To describe mental health staff experiences of giving support to prevent type 2 diabetes mellitus (DM) among people with psychosis in community psychiatry.	Qualitative exploratory study	Semi-structured interviews	Community mental health services	12 community healthcare staff	Community mental healthcare staff	Interviews were conducted in the context of antipsychotics increasing risks of cardiovascular and metabolic comorbidities
Hultsjö, 2013; Sweden [21]	To explore and analyse mental healthcare staff’s (MHCS) knowledge and experiences of diabetes care for persons with psychosis	Qualitative exploratory study	Semi-structured interviews	Unclear	12 mental health care staff	Mental health care staff	Participants described barriers to monitoring and management of cardiovascular and metabolic side effects
Hyland, 2003; Australia [31]	To examine the attitudes and practices of case managers working in Area Mental Health Services (AMHS) towards the physical health of people with chronic mental illness.	Mixed method; cross sectional study and interview	Focus group interviews (4 groups)	Multicentre (4 mental health clinics)	111 case managers working in community mental health	Community mental health case managers	Participants described barriers to monitoring and management of cardiovascular and metabolic side effects
Mangurian, 2013; USA [22]	To examined primary care providers’ beliefs about the roles that primary care providers and psychiatrists should play in metabolic monitoring and treatment of metabolic abnormalities among people with severe mental illness.	Cross sectional study	survey	Urban safety net clinic	214 primary health care providers from 23 public community healthClinics.	Primary health care providers	Participants described barriers to monitoring and management of cardiovascular and metabolic side effects amongst their patients
McDonell, 2011; USA [25]	To assess the relative importance of patient, provider, and systemic barriers to metabolic syndrome management for persons with severe mental illness	Cross sectional study	Survey	Not specific	68 medical, mental health, and other stakeholders who care for patients with severe mental illness.	Wide range of healthcare professionals.	All health care providers who participated were involved in delivery of care for adults prescribed antipsychotics
Mwebe, 2017;UK [11]	To explore nurses’ views of their role in the screening and monitoring of the physical care needs of people with serious mental illness in a mental health service provider	Qualitative exploratory study	Semi-structured interviews	Mental health inpatient centre	10 mental health nurses	Mental health nurses	Nurses described barriers to monitoring and management of cardiovascular and metabolic side effects amongst their patients
Robson, 2013; UK [23]	To examine mental health nurses’ attitudes to physical health care and explore associations with their practice and training.	Cross sectional study	Survey	National Health Service (NHS) Mental Health Trust	Sample of 585 qualified mental health nurses	Mental healthcare staff (Mental health nurses)	Participants described barriers to monitoring and management of cardiovascular and metabolic side effects amongst their patients
Wheeler, 2010;New Zeeland [28]	To invest health practitioners’ views on their role in assessing and managing their clients’ cardiovascular risk profile. We also sought to explore the practitioners’ perceptions of barriers and solutions for the management of cardiovascular risk in people with mental illness.	Qualitative study	Semi-structured interview	Unclear	Sample of 9 participants	Healthcare professionals (psychiatrists/psychiatric trainees, general practitioners, nurse specialists, mental health pharmacists, and consumer advisors.	Healthcare professionals described barriers to monitoring and management of cardiovascular and metabolic side effects

### Quality of the included studies

The assessment was based on the four critical appraisal categories of qualitative evidence): credibility, transferability, dependability, and confirmability. Of the 10 elements that are presented by the assessment tool (JBI), most studies fulfilled at least 6 criteria. Most studies met quality indicators related to the congruity of the selected method to answer the research question, rigour sampling and data collection method, and representation of participants’ voice. Confirmability across the studies was generally weak as none of the included studies failed to provide information regarding the potential influence of the researcher on the study results (Electronic supplementary material 3).

### The emerged themes

A total of 40 themes were extracted, then inductively categorised into nine categories and further into two synthesised findings extrinsic and intrinsic factors. A detailed description of the identified themes and corresponding sub-themes is reported in Table 3. Key categories and themes are described below.

**Table 3:** Themes, Categories and synthesised findings in relation to barriers to monitoring and management of cardiovascular and metabolic health of patients prescribed antipsychotic medicines

Themes	Category	Synthesised findings
Social context and support [14–16, 27, 29]		Extrinsic factors
Lack of support from family/friends [12, 13, 21, 24]		
Poor communication/ coordination between different health care providers /sectors [11, 12, 21, 22, 26, 28, 31]	Provider and healthcare system factors	
Insufficient physician time [14, 20, 22, 23, 25, 26, 29]		
Fragmentation of care [12, 20, 25, 27]		
Patient- health-care professional relationship and communication [12, 21]		
Issue of lack of clarity for responsibility for conducting physical health care [20, 21]		
Lack of resources to manage healthy life style [11, 12, 17, 24, 27, 28]	Lack of resources	
Lack of equipment or suitable space [26–28]		
Staff turnover [26, 27]		
Lack of qualified staff to manage physical health issues [25]		
Primary care providers are not paid enough [25]		
Lack of insurance [21]		
Cost of running interventions or groups [26]		
Lack of access to qualified psychiatric follow-up [16, 22, 25]	The Intervention site and the intervention characteristics	
Intensity of the applied intervention [15, 19]		
Leadership is not making managing physical health issues among patients with SMI a priority [21, 25, 26]		
Difficulty traveling to specialised service [26]		
Transportation problems [12]		
Poor patient attendance [26]		
Social context and support [14–16, 27, 29]	Family and community factor	
Lack of support from family/friends [12, 13, 21, 24]		
Mental health condition related disabilities [11, 13, 17, 19, 25, 29–31]	Mental health condition as a barrier	Intrinsic Factors
Severity of psychotic symptoms [17, 21, 22, 24, 25, 30, 31]		
Consequences associated with the side effects of the antipsychotic medications [19, 21, 25, 29]		
Stigma/Isolation/estrangement [12, 14, 20, 27, 28]		
Motivation level among participants regarding general health issues [16–18, 21, 23–27]	Psychological factors	
Participants views and behaviour toward specific intervention [15, 23, 26, 27, 29]		
Participants’ views and behaviour regarding screening, monitoring of general health issues [11, 26, 31]		
Stress [12, 18, 26]		
Participants’ knowledge about healthy lifestyle [21, 22, 26, 30]	knowledge and skills	
Participants’ knowledge about the medicines side effects [23, 27]		
Lack of knowledge and training among health-care providers to manage physical health issues [14, 19, 20, 25, 26, 28]		
Functional limitation [12, 24]	multiple-physical comorbidities	
Negative effects of certain conditions e.g. pain [17]		
Non-concordance with drug therapy [26]	Compliance issues	
Social context and support [14–16, 27, 29]	Family and community factor	
Lack of support from family/friends [12, 13, 21, 24]		

### Extrinsic factors

This theme includes factors related to the patients’ community and their surrounding [12–17, 19–26, 28, 31].

#### Provider and health-care system factors

Care coordination and teamwork between different sectors and health-care professionals were deemed essential for timely screening and monitoring [11, 21, 22, 31]. According to one study, [31] low rate of physical health examination in patients with psychosis was attributed to the lack of care delivery integration between general practitioners and psychiatrists. Similarly, primary care providers complained about the lack of collaboration between health care professionals from different sectors which resulted in difficulties to refer patients to specialised care [22].

Managing general health conditions in patients with mental health conditions such as diabetes were not considered as a priority in some primary care settings [21, 25, 26]. Effective communication between health-care providers was cited as vital [12, 26, 28]. For instance, general practitioners pointed out that communication difficulties often occurred during the transfer of care across sectors (e.g. primary to secondary care), and as a result, essential information including test results was often missed [28].

Time constraints was a main restriction for effective interaction between health professionals and their patients [14, 20, 22, 23, 25, 26, 28]. Patients complained about their inability to discuss their conditions with their health-care professionals due to short consultation time [22]. Similarly, mental health nurses were concerned regarding their inability to check patients’ physical health due to their heavy work schedule [20].

The positive relationship between patients and their health care providers were reported to be helpful for better health outcomes [11, 15]. The patient-provider relationship was reported to be indirectly influenced by the behaviour of health care providers at the time of consultation. Patients reported that the disinterest they observed from the health-care professionals resulted in a lack of trust between the two parties. Consequently, patients may hesitate to share their physical issues with their providers [12, 26].

#### Lack of resources

Health professionals considered resource limitations and restrictions imposed by funding bodies such as national health systems and insurance companies as barriers that hinder their ability to manage the physical health of patients [11, 20, 22, 25–27, 31].

The lack of financial support from organisational bodies appeared to affect the quality of care delivered to manage physical health issues in patients with mental health conditions [11, 13, 20, 22, 25, 26, 28]. This factor contributed to several issues mainly lack facilities and equipment to screen physical health issues; lack of professional training necessary to manage general health conditions (e.g. diabetes) [20, 26, 27]. Participants, particularly health care providers in mental health clinics, reported difficulties to collect patients’ blood sample due to the unavailability of laboratory services at the clinic. Consequently, patients had to be referred to another centre to carry out the analysis [27]. Similarly, mental health nurses pointed out that lack of economic support was a major factor that hindered embedding physical health screening and monitoring into their clinic [11, 20, 26].

Notably, insufficient financial support appeared to impair the performance of staff working in mental health services [18, 28, 29]. The increased concerns regarding the notable increase in resignation rate among health care providers, as they were not satisfied with the increased workload and decreased payment [25–27]. Patients shared similar views regarding the costs of the available services to manage their physical health [11, 17, 24, 27, 28]. Patients participated in lifestyle/weight loss interventions programs complained about their inability to afford the money to buy healthy food [27]. Similarly, health-care professionals highlighted the poor financial status of the patients as a barrier for their compliance with cardiovascular screening [24].

Further concerns raised regarding the availably of competent staff to manage physical health issues in patients with mental health conditions [26]. Both health-care professionals and patients agreed on the importance of professional training as it was seen as a facilitator that enables the providers to manage comorbid general health conditions [14, 19, 20, 25, 26, 28]. As a result, health-care professionals in mental health-care services had to limit their efforts and focus on managing mental health conditions only despite co-existing morbidities such as diabetes [25, 27]. Unavailability of the appointments within general practices was also cited as a barrier [25, 26].

#### The intervention site and the intervention characteristics

The subcategories related to this theme reflected factors related to the intervention setting and the characteristics of the applied intervention (e.g. physical health management programmes). Lack of access to specialist health-care professionals or services to check physical health issues among patients was indirectly cited as a barrier by both patients and health-care professionals [12, 16, 21, 22, 25, 26].

Both health-care professionals and patients agreed that scarce facilities for monitoring physical health (e.g. general practices), could result in travelling difficulties among patients and hence irregular cardiovascular follow-up [12, 26]. The intensity of the exercise sessions was highlighted as a barrier that prevents patients from regularly participating in exercise-based interventions [15].

#### Patients’ family and community factors

This theme comprises factors that are related to patients-family/community relationship. Social support was vital for successful management of co- morbidities in patients with psychosis [11, 13, 15, 21, 24]. Many patients agreed that group-based interventions had a positive impact on their relationship as it contributed to new friendships [11, 16, 21]. Furthermore, involving family members in lifestyle modifying interventions deemed to have positive and long-term impacts for patients and their families [15, 21].

However, the same experience was not always associated with positive results for others [15, 29]. Anxiety resulting from being surrounded by “strangers” represented additional burden for some patients and hence hindered their participation in group-based interventions [29]. A similar effect was reported by another study, [15] which highlighted that participants (patients) in group-based exercise tended to compare their achievement with their peers which consequently resulted in their withdrawal from the sessions.

### Intrinsic factors (patients related factors)

Mental health condition, psychological factors, participants’ knowledge and skills and existing co-morbidities were the key barriers to undergoing screening, monitoring and managing cardiovascular and metabolic issues in patients with mental health problems (Table 3).

#### Mental health condition as a factor

This theme and its related sub-themes: disabilities associated with the psychotic disorder, consequences of the antipsychotic medications regarding participation in physical health activities, the severity of psychiatric illness and stigma/isolation/estrangement were recognised by both patients and care providers that negatively affect screening, monitoring and management of cardiovascular co-morbidities [11–14, 17, 19, 21, 24, 25, 29, 31].

Severity of psychotic symptoms; adverse effects of psychotropic medicines were recognised by both patients and care providers that negatively affect screening, monitoring and management of cardiovascular co-morbidities [11, 13, 17, 19, 25, 29–31]. A major difficulty, from health-care professionals’ perspective, was the aggressive behaviour and depression resulting from the psychotic symptoms [22, 25]. Mental health nurses spoke of the need to prescribe tranquilisers that would manage aggressive behaviour among patients with mental health conditions which would enable better screening and monitoring [19]. Additionally, patients reported that some psychotic symptoms, mainly depression prevented them from actively participating in physical activities [13, 25].

The severity of psychotic symptoms represented another barrier for screening and management of cardiovascular co-morbidities for both patients and health care providers [17, 21, 22, 24, 25, 30, 31]. According to one study, [22], around 35% of participants (community health-care professionals) agreed that the more severe mental health conditions, the more difficult to conduct screening of co-morbidities. Similarly, there was a notable lack of cooperation among patients toward simple procedures, including giving blood samples [19].

Both health-care professionals and patients expressed their concerns regarding some side effects of psychotic medicines which interfere with patients’ engagement in physical activities [19, 21, 29, 30]. Health professionals stated that the sedative side effects of some psychotropic medications could make it difficult for patients to participate in exercise-based programmes [19, 29]. Findings of a study exploring diabetic care experience and knowledge among mental health-care staffs showed that low rate of patients’ attendance to diabetes appointments was associated with the hangover symptoms of psychotic medicines mainly drowsiness [21].

“Mental health stigma” or having mental health conditions was highlighted in several studies as a patient-related barrier to effective screening and management of cardiovascular co-morbidities [12, 14, 17, 24, 25]. The effect of stigma was reflected as difficulties raised from the distorted image of being mentally ill, which can affect a patient’s interaction with others (e.g. family). For example, patients believed that having mental health conditions compromised their relationship with their families by assuming that they are incapable of dealing with their daily life challenges. Consequently, patients felt isolated and estranged among their own families and hence hesitated to ask for help when needed [12].

#### Psychological factors

Intrinsic psychological factors such as participants’ views, feelings, behaviour and attitude were the most commonly cited factors. Patients with mental health conditions seemed to hold various views and attitudes toward their conditions which could affect their motivation to receive treatment of any kind. Patients highlighted negative attitudes and views such as hopelessness, low self-esteem/motivation as major barriers for receiving cardiovascular or metabolic care [11, 12, 15, 16, 18, 21, 23, 27, 29, 31].

Motivation was perceived as a driving force for adopting healthy lifestyle among patients with mental health conditions [16, 18, 21, 23, 27]. Patients’ optimism of a good healthy life was strongly associated with the intentions to participate in physical activity programs [24]. On the contrary, lack of motivation among patients with mental health conditions was a major obstacle that prevented adopting healthy life behaviour [21, 23, 24]. Particularly, low expectation associated with mental health conditions acted as a barrier for some patients to initiate physical activities.

Participants including health care providers and patients, agreed that patients’ negative views towards specific interventions that are intended to manage cardiovascular side effects (e.g. exercise-based interventions) could affect their adherence or participation in the program, mainly due to general dislike or disinterest [23, 26, 31]. Previous unsuccessful experiences shaped participants’ views towards particular intervention such as exercise [15, 29]. For instance, patients, who underwent a failed experience, felt distressed when their expectations were not met, and hence, they would withdraw from the program [15].

Generally, patients expressed unfavourable attitude toward physical health screening and monitoring. In several studies, patients tended to ignore their physical health due to several reasons including lack of concerns over their general health [18, 26, 31] additional to lack sense of worth [31].

Some studied showed that patients’ excessive concerns regarding their general health represented an additional burden that would hinder their abilities to manage their physical health issues [12, 18, 26].

#### Knowledge and skills

Health-care providers tend to agree on the importance of having solid knowledge about cardiovascular and metabolic risks associated with antipsychotic drugs and the necessary skills to manage them [19, 21, 28]. Health-care professionals expressed their concerns about the difficulty to identify patients in need for cardio-metabolic monitoring and hence their inabilities to manage those patients [21, 25, 26, 30] properly. For example, one study aimed to explore mental health-care staff’s knowledge and experiences to manage diabetes in patients with psychosis identified that key factors affecting diabetic care delivered by mental health-care staff was having knowledge about diabetes as it increased their confidence to deal with susceptible diabetic cases among patients.

Besides, participants’ knowledge about positive lifestyle habits such as a healthy diet was cited as a facilitator for patient’s participation in weight management programs [21, 22, 26, 30]. Patients’ felt that having solid knowledge about the nutritional facts was important as it helped them adopt healthy lifestyle [13, 18]. Patients’ knowledge about the metabolic side effects of antipsychotic medicines increased their awareness of their general health and hence motivated those to take actions (participate in physical health activity programs) [13]. However, knowledge did not necessarily associate with positive health outcomes [13, 23]. Robson et al. [23] found that around 21.7% of the respondents (*n* = 125/577) agreed that patients’ knowledge about the side effects of their psychotic medicines was associated with low adherence.

#### Physical co-morbidities in patients with mental health conditions

For some patients, managing multiple-comorbidities was stressful and difficult [11, 17, 24]. For instance, a complex treatment regimen for both mental health and chronic conditions (e.g. diabetes) caused difficulties among patients due to the concurrent demands of competing co-morbidities [11]. Also, physical complications such as chronic pain and fatigue resulted in difficulties for patients during exercise-based interventions [11].

## Discussion

To the best of our knowledge, this is the first systematic review of contributory factors for effective monitoring and management of cardiovascular and metabolic co-morbidities in patients taking antipsychotic medicines. This review identified a number of potential key factors which affect the screening and management of physical health issues, namely cardiovascular and metabolic side effects of antipsychotic drugs.

Themes concerning mental health conditions as identified in this study can be related to the stigma model proposed by Knaak et al. [32] and Ahmedani et al. [33]. Personal stigma refers to the self-perception of stigma. Interpersonal stigma refers to the stigmatised attitudes/ beliefs towards individuals with mental health conditions. Structural stigma refers to the discrimination in health-care services that patients may encounter. Patients may lack confidence and hesitate to share issues related to their physical health. Evidence on studies associated with mental health- stigma in health-care [32] showed that even well-trained professionals working in mental health disciplines are subjected to interpersonal mental health stigma. This can result in a lack of mutual trust between patients and health-care professionals and hence affect information sharing between the involved parties. Anti-stigma intervention for HCPs, such as the ‘targeting the roots of health-care provider stigma’, [34] can be useful. This model requires improving: the ability of health-care professionals to manage and cope with their emotions when working with patients in challenging situations; improving competence and confidence of staff; and addressing the lack of awareness of one’s prejudices.

This systematic review shows that symptoms associated with severe mental health conditions often make health-care professionals and patients engage in physical health monitoring. Particularly, the aggression and depressive symptoms associated with these conditions alongside the sedative effects of some antipsychotic medicines presents as a barrier to effective monitoring and management. Appropriate choice of drug therapy is imperative in managing symptoms of severe mental health that can enable better communication between patient and health-care professionals [35]. Family and carer involvement is imperative in the process. Peer-led support groups have also been shown to be effective in improving hope, recovery and empowerment [36]. There is a scope for such interventions to be tested in improving physical health monitoring programmes.

The organisational and system-related barriers to the monitoring of physical health amongst patients taking antipsychotic medicines require urgent attention. Fragmentation of care was identified to be a key barrier. Fragmented responsibilities of specialist mental health services, primary care including community psychiatric clinics were shown to have led to confusion amongst health-care professionals with regards to the roles and responsibilities in monitoring physical health and the referral process. While NICE guideline in England provides clear remit for primary care in monitoring physical health, the practice seems to be suboptimal. In addition, there is a need to improve on the knowledge of mental health service providers on physical health and monitoring. Furthermore, the studies demonstrated lack of resources and funding that constrained their responsibilities. In the UK, the one appointment one problem culture often contributed to the problem. The resource was also a patient-related barrier as studies described a lack of means for the patients to afford healthy eating and exercise.

This review suggests that having solid and trusting relationship was vital for patients with mental health conditions as it promotes better health outcomes. The review highlighted a pattern of hesitation to seek help among patients. Patients with mental health conditions often feel isolated from the community. Subsequently, they tend to be more independent and become their own counsellors [37], which cause more isolation. Therefore, considering the psych-social aspects of mental health conditions by involving the family and friends in the management process is vital.

### Strengths and limitations

The current review has several limitations. Some of the included studies in this review did not recruit patient participants based on their use of antipsychotic drugs. However during full text screening, it was decided to include the studies where participant accounts of barriers to follow up and monitoring of cardiovascular side effects of antipsychotic drugs were presented in the study results. Moreover, the included studies originated mainly from the Western countries, which limit the generalisability of our findings.

Despite the limitations, this review provides an insight into potential factors influencing screening and management of cardiovascular and metabolic side effects of antipsychotics. The review employed an inductive thematic synthesis of qualitative studies of participants’ perceptions from various backgrounds. Using such an approach provides reflexivity as it involves exploring the available evidence from participants’ perspectives. Besides, our flexibility in the study inclusion criteria needs to be considered when generalising the study findings. Furthermore, the methodological quality of included studies was assessed using Joanna Briggs (BJI) assessment tools, which provides uniform and structured evaluation of the studies.

### Implications for practice

Despite the evidence and guidelines that support the importance management of cardiovascular and metabolic side effects of antipsychotics, regular assessment of the implementation of such guideline is imperative. Participants of the studies included in the systematic review prefer integrated models of monitoring and management of both physical and mental health, and there is a need to minimise fragmentation of care. This can be facilitated by addressing the contributory factors to monitoring and management of the side effects as identified in this systematic review.

Individualised interventions to tailor the needs of the patients shown to minimise barriers to participation in exercise-based interventions. Patients with severe mental health conditions often face barriers to managing their physical health due to negative symptoms and cognitive impairment. Therefore, knowledge-based interventions alone are not adequate. Persistent and sustainable involvement is necessary. Peer-led support network is known to be important in promoting engagement in physical exercise. These are particularly important where patients lack family and friends support. Funding bodies and stakeholders must understand the needs of their employers. This can be fulfilled by promoting professional development within the organisation.

Previously, suggestions have been made around clinical, particularly nursing staff being trained to manage both physical and mental health issues in order to provide person-centred care. Previous literature has shown that nurses are generally in favour of physical health care as part of their role [38]. However, those without the background of generalist nurse training were less comfortable about their expertise. Appropriate reimbursements should be provided to uptake additional roles in community mental health and specialist psychiatric units.

### Future directions

Currently, this review suggests that numerous patients prescribed antipsychotics are not properly monitored, counselled or managed for cardiovascular and metabolic side effects. The limited number of the available studies addressed factors barriers/facilitators for screening and management of antipsychotics associated cardiovascular and metabolic issues. This suggests poor empirical evidence which underpins current information practice in contributory factors for the management of cardiovascular and metabolic side effects of antipsychotic medicines. Further research is required to address contributory factors for sub-optimal management of cardiovascular and metabolic side effects of antipsychotics from the perspectives of patients, health-care professionals, family and carers of patients with severe mental health problems.

While this review focused on barriers for screening and management of cardiovascular co-morbidities in patients with mental health conditions, future research should be directed to elicit facilitators to neutralise those barriers. Furthermore, future research should target stigma facilitators at different levels. Further studies on health-care professionals’ stigma are essential to address all dimensions of stigma across different levels. Obtaining the views of wider stakeholders would provide further insight into the barriers and facilitators to resource allocations between mental and general health sectors. Multi-morbidity including poor mental, physical health and substance misuse and poor access to services can lead to homelessness and social disparities [39–42]. There is a need to develop innovative services delivery models, such as outreach programmes to support patients with severe mental health [43–45] and evaluate their impact on health and quality-of-life outcomes.

## Conclusions

This study demonstrates that barriers to monitoring, counselling and management of cardiovascular and metabolic health of patients taking antipsychotic medicines are multidimensional. Knowledge-based interventions alone will be insufficient in improving the practice. There is a need to address the fragmentation of care, lack of resources to address co-morbidity in clinical consultations and stigma in the health-care setting. There is a scope to develop, implement and evaluate peer and family support-based interventions in improving the practice.

## Supplementary Information

**Additional file 1: Supplementary material 1**. Literature search strategy. **Supplementary material 2**. PRISMA check list for the present review**. Supplementary material 3**. Quality assessment of the selected studies.

## Data Availability

All data generated or analysed during this study are included in this published article [and its supplementary information files].

## Abbreviations

ADA: American Diabetes Association
APA: American Psychiatric Association
C: Credible
CRD: Centre for Review and Dissemination
ECG: Electrocardiogram
FDA: Food and Drug Administration
JBI-QARI: Joanna Briggs Institute Assessment and Review Instrument
NICE: National Institute for Health and Care Excellence
PRISMA: Preferred reporting items for systematic review and meta-analysis
PRISMA-P: Preferred reporting items for systematic review and meta-analysis- Protocol version
U: Unequivocal
US: Unsupported
