# Supplementary material for: Barriers to monitoring and management of cardiovascular and metabolic health of patients prescribed antipsychotic drugs: a systematic review
Source: BMC Psychiatry. 2020 Dec 4;20:581. doi: 10.1186/s12888-020-02990-6 (PMC7718699; doi:10.1186/s12888-020-02990-6)
Supplement: Supplementary file 1 — Additional file 1: Supplementary material 1. Literature search strategy. Supplementary material 2. PRISMA check list for the present review. Supplementary material 3. Quality assessment of the selected studies. [file 12888_2020_2990_MOESM1_ESM.doc]

**Supplementary material 1: Literature search strategy**

Medline 09 Oct 2019

Population and (condition/context)

1. Schizophrenia/ or Psychotic Disorders/
2. psychotic symptom*.mp. or Hallucinations/
3. Mental Disorders/dt, mo [Drug Therapy, Mortality]
4. Antipsychotic Agents/ae [Adverse Effects]
5. ANTIPSYCHOTIC drug*.mp.
6. Antipsychotic medication*.mp.
7. First generation antipsychotic*.mp.
8. Second generation antipsychotic*.mp.
9. Neuroleptic drug*.mp.
10. Neuroleptic agent*.mp.
11. Neuroleptic medication*.mp.
12. Antipsychotic medicine*.mp.
13. Depressive Disorder/dt, mo [Drug Therapy, Mortality]
14. DEPRESSION/dt, mo [Drug Therapy, Mortality]
15. Antidepressive Agents, Second-Generation/ or Serotonin Uptake Inhibitors/ or Antidepressive Agents/ or antidepressant*.mp. or Antidepressive Agents, Tricyclic/
16. selective serotonin re-uptake inhibitor*.mp.
17. tricyclic related antidepressant*.mp.
18. (Serotonin and Noradrenaline Reuptake Inhibitor*).mp. [mp=ti, ab, hw, tn, ot, dm, mf, dv, kw, fx, dq, nm, kf, ox, px, rx, ui, sy]
19. Cardiovascular Diseases/di, ep, pc [Diagnosis, Epidemiology, Prevention & Control]
20. Heart Diseases/co, di, ep, pc [Complications, Diagnosis, Epidemiology, Prevention & Control]
21. Coronary Disease/ or Heart Failure/ or cardiac complication*.mp. or Myocardial Ischemia/ or Myocardial Infarction/
22. cardiovascular complication*.mp.
23. Cardiovascular effect*.mp.
24. Cardiac effect*.mp.
25. Cardiovascular side effect*.mp.
26. Hypertension/ or Obesity/ or Cardiovascular risk factor*.mp. or Diabetes Mellitus, Type 2/
27. Metabolic Syndrome/di, pc [Diagnosis, Prevention & Control]
28. Insulin Resistance/ or Metabolic disturbance*.mp.
29. Glucose Intolerance/di, pc [Diagnosis, Prevention & Control]
30. (glucose intolerance adj4 schizophrenia).mp. [mp=ti, ab, hw, tn, ot, dm, mf, dv, kw, fx, dq, nm, kf, ox, px, rx, ui, sy]
31. (glucose intolerance adj4 depression).mp. [mp=ti, ab, hw, tn, ot, dm, mf, dv, kw, fx, dq, nm, kf, ox, px, rx, ui, sy]
32. Metabolic dysregulation*.mp.
33. cardiometabolic*.mp.
34. cardiovascular risk.mp.
35. cardiovascular event*.mp.
36. major adverse cardiovascular event*.mp.
37. 1 or 2 or 3 or 4 or 5 or 6 or 7 or 8 or 9 or 10 or 11 or 12 or 13 or 14 or 15 or 16 or 17 or 18
38. 19 or 20 or 21 or 22 or 23 or 24 or 25 or 26 or 27 or 28 or 29 or 30 or 31 or 32 or 33 or 34 or 35 or 36
39. 37 and 38
40. perspective*.mp.
41. experience*.mp.
42. *PERCEPTION/
43. view.mp.
44. believe.mp.
45. *PATIENT PREFERENCE/ or preference*.mp.
46. incentive*.mp.
47. barrier*.mp.
48. *"Surveys and Questionnaires"/ or facilitator*.mp.
49. *QUALITATIVE RESEARCH/
50. "QUALITATIVE STUDY".mp. [mp=ti, ab, hw, tn, ot, dm, mf, dv, kw, fx, dq, nm, kf, ox, px, rx, an, ui, sy]
51. "content analysis"/ or "thematic analysis"/ or phenomenology.mp. [mp=ti, ab, hw, tn, ot, dm, mf, dv, kw, fx, dq, nm, kf, ox, px, rx, an, ui, sy]
52. interview*.mp. [mp=ti, ab, hw, tn, ot, dm, mf, dv, kw, fx, dq, nm, kf, ox, px, rx, an, ui, sy]
53. "indepth interview*".mp. [mp=ti, ab, hw, tn, ot, dm, mf, dv, kw, fx, dq, nm, kf, ox, px, rx, an, ui, sy]
54. "focus group*".mp. [mp=ti, ab, hw, tn, ot, dm, mf, dv, kw, fx, dq, nm, kf, ox, px, rx, an, ui, sy]
55. 40 or 41 or 42 or 43 or 44 or 45 or 46 or 47 or 48 or 49 or 50 or 51 or 52 or 53 or 54
56. 39 and 55
57. limit 56 to english language
58. limit 57 to yr="2006 -Current"
59. limit 58 to humans

**Supplementary material 2: PRISMA check list for the present review**

| **Section/topic** | **#** | **Checklist item** | **Reported on section** |
| --- | --- | --- | --- |
| **TITLE** | | |  |
| Title | 1 | Identify the report as a systematic review, meta-analysis, or both. | Title page |
| **ABSTRACT** | | |  |
| Structured summary | 2 | Provide a structured summary including, as applicable: background; objectives; data sources; study eligibility criteria, participants, and interventions; study appraisal and synthesis methods; results; limitations; conclusions and implications of key findings; systematic review registration number. | Abstract page |
| **INTRODUCTION** | | |  |
| Rationale | 3 | Describe the rationale for the review in the context of what is already known. | Background |
| Objectives | 4 | Provide an explicit statement of questions being addressed with reference to participants, interventions, comparisons, outcomes, and study design (PICOS). | Background |
| **METHODS** | | |  |
| Protocol and registration | 5 | Indicate if a review protocol exists, if and where it can be accessed (e.g., Web address), and, if available, provide registration information including registration number. | Title page and Methodology |
| Eligibility criteria | 6 | Specify study characteristics (e.g., PICOS, length of follow-up) and report characteristics (e.g., years considered, language, publication status) used as criteria for eligibility, giving rationale. | Methodology |
| Information sources | 7 | Describe all information sources (e.g., databases with dates of coverage, contact with study authors to identify additional studies) in the search and date last searched. | Methodology |
| Search | 8 | Present full electronic search strategy for at least one database, including any limits used, such that it could be repeated. | Supplement A |
| Study selection | 9 | State the process for selecting studies (i.e., screening, eligibility, included in systematic review, and, if applicable, included in the meta-analysis). | Methodology |
| Data collection process | 10 | Describe method of data extraction from reports (e.g., piloted forms, independently, in duplicate) and any processes for obtaining and confirming data from investigators. | Methodology |
| Data items | 11 | List and define all variables for which data were sought (e.g., PICOS, funding sources) and any assumptions and simplifications made. | Methodology |
| Risk of bias in individual studies | 12 | Describe methods used for assessing risk of bias of individual studies (including specification of whether this was done at the study or outcome level), and how this information is to be used in any data synthesis. | Methodology |
| Summary measures | 13 | State the principal summary measures (e.g., risk ratio, difference in means). | NA |
| Synthesis of results | 14 | Describe the methods of handling data and combining results of studies, if done, including measures of consistency (e.g., I^2^) for each meta-analysis. | Methodology |

Page 1 of 2

| **Section/topic** | **#** | **Checklist item** | **Reported on page #** |
| --- | --- | --- | --- |
| Risk of bias across studies | 15 | Specify any assessment of risk of bias that may affect the cumulative evidence (e.g., publication bias, selective reporting within studies). | Methodology |
| Additional analyses | 16 | Describe methods of additional analyses (e.g., sensitivity or subgroup analyses, meta-regression), if done, indicating which were pre-specified. | NA |
| **RESULTS** | | |  |
| Study selection | 17 | Give numbers of studies screened, assessed for eligibility, and included in the review, with reasons for exclusions at each stage, ideally with a flow diagram. | Results |
| Study characteristics | 18 | For each study, present characteristics for which data were extracted (e.g., study size, PICOS, follow-up period) and provide the citations. | Results |
| Risk of bias within studies | 19 | Present data on risk of bias of each study and, if available, any outcome level assessment (see item 12). | Results |
| Results of individual studies | 20 | For all outcomes considered (benefits or harms), present, for each study: (a) simple summary data for each intervention group (b) effect estimates and confidence intervals, ideally with a forest plot. | NA |
| Synthesis of results | 21 | Present results of each meta-analysis done, including confidence intervals and measures of consistency. | Results |
| Risk of bias across studies | 22 | Present results of any assessment of risk of bias across studies (see Item 15). | NA |
| Additional analysis | 23 | Give results of additional analyses, if done (e.g., sensitivity or subgroup analyses, meta-regression [see Item 16]). | NA |
| **DISCUSSION** | | |  |
| Summary of evidence | 24 | Summarize the main findings including the strength of evidence for each main outcome; consider their relevance to key groups (e.g., healthcare providers, users, and policy makers). | Discussion |
| Limitations | 25 | Discuss limitations at study and outcome level (e.g., risk of bias), and at review-level (e.g., incomplete retrieval of identified research, reporting bias). | Discussion |
| Conclusions | 26 | Provide a general interpretation of the results in the context of other evidence, and implications for future research. | Discussion |
| **FUNDING** | | |  |
| Funding | 27 | Describe sources of funding for the systematic review and other support (e.g., supply of data); role of funders for the systematic review. | NA |

*From:*  Moher D, Liberati A, Tetzlaff J, Altman DG, The PRISMA Group (2009). Preferred Reporting Items for Systematic Reviews and Meta-Analyses: The PRISMA Statement. PLoS Med 6(7): e1000097. doi:10.1371/journal.pmed1000097

For more information, visit: **www.prisma-statement.org**.

Page 2 of 2

**Supplementary material 3: Quality assessment of the selected studies**

| Criteria | Mwebe, 2017 [8] | Blixen, 2016 [9] | Yarborough ,2016 [10] | Kristiansen ,2015 [11] | Wärdig, 2015 [12] | Graham ,2014 [13] | Rastad, ,2014 [14] | Pearsal, ,2014 [15] | Bergqvist, 2013 [16] | Happell ,2013 [17] | Hultsjö ,2013 [18] |
| --- | --- | --- | --- | --- | --- | --- | --- | --- | --- | --- | --- |
| 1. Is there congruity between the stated philosophical perspective and the research methodology? | Yes | Unclear | Yes | Unclear | Yes | Yes | Yes | Yes | Yes | Yes | Yes |
| 2. Is there congruity between the research methodology and the research question or objectives? | Yes | Yes | Yes | Yes | Yes | Yes | Yes | Yes | Yes | Yes | Yes |
| 3. Is there congruity between the research methodology and the methods used to collect data? | Yes | Yes | Yes | Yes | Yes | Yes | Yes | Yes | Yes | Yes | Yes |
| 4. Is there congruity between the research methodology and the representation and analysis of data? | Yes | Unclear | Yes | Yes | Yes | Yes | Yes | Yes | Yes | Yes | Yes |
| 5. Is there congruity between the research methodology and the interpretation of results? | Yes | Yes | Yes | Yes | No | Yes | Yes | Yes | Yes | Yes | Yes |
| 6. Is there a statement locating the researcher culturally or theoretically? | Unclear | Unclear | Unclear | Unclear | Unclear | Unclear | Unclear | Unclear | Unclear | Unclear | Unclear |
| 7. Is the influence of the researcher on the research, and vice- versa, addressed? | Unclear | Unclear | Unclear | Unclear | Unclear | Unclear | Unclear | Unclear | Unclear | Unclear | Unclear |
| 8. Are participants, and their voices, adequately represented? | Yes | Yes | Yes | Yes | Yes | Yes | Yes | Yes | Yes | Yes | Yes |
| 9. Is the research ethical according to current criteria or, for recent studies, and is there evidence of ethical approval by an appropriate body? | Yes | Yes | Yes | Yes | Yes | Yes | Yes | Yes | Yes | Yes | Yes |
| 10. Do the conclusions drawn in the research report flow from the analysis, or interpretation, of the data | Yes | Yes | Yes | Yes | Yes | Yes | Yes | Yes | Yes | Yes | Yes |

| Criteria | Mangurian , 2013 [19] | Robson ,2013 [20] | Hultsjö ,2012 [21] | Vandyk, 2012 [22] | McDonell , 2011 [23] | Pitman ,2011 [24] | Yarborough ,2011 [25] | Wheeler ,2010 [26] | Johnstone ,2009 [27] | McDevitt ,2006 [28] | Hyland ,2003 [29] |
| --- | --- | --- | --- | --- | --- | --- | --- | --- | --- | --- | --- |
| 1. Is there congruity between the stated philosophical perspective and the research methodology? | Unclear | Yes | Yes | Yes | No | Unclear | Unclear | Yes | Unclear | Unclear | Unclear |
| 2. Is there congruity between the research methodology and the research question or objectives? | Yes | Yes | Yes | Yes | No | Yes | Yes | Yes | Yes | Yes | No |
| 3. Is there congruity between the research methodology and the methods used to collect data? | Yes | No | Yes | Yes | No | Yes | Yes | Yes | Yes | Yes | No |
| 4. Is there congruity between the research methodology and the representation and analysis of data? | Yes | Yes | Yes | Yes | No | Yes | Yes | Yes | Yes | Yes | No |
| 5. Is there congruity between the research methodology and the interpretation of results? | Yes | Yes | Yes | Yes | No | Yes | Yes | Yes | Yes | Yes | Yes |
| 6. Is there a statement locating the researcher culturally or theoretically? | Unclear | Unclear | Unclear | Unclear | Unclear | Unclear | Unclear | Unclear | Unclear | Unclear | Unclear |
| 7. Is the influence of the researcher on the research, and vice- versa, addressed? | Yes | Unclear | Unclear | Unclear | Unclear | Unclear | Unclear | Unclear | Unclear | Unclear | Unclear |
| 8. Are participants, and their voices, adequately represented? | Yes | No | Yes | Yes | NA | Yes | No | Yes | Yes | Yes | Yes |
| 9. Is the research ethical according to current criteria or, for recent studies, and is there evidence of ethical approval by an appropriate body? | Yes | Yes | Yes | Yes | Yes | Yes | Yes | Yes | Yes | Yes | Yes |
| 10. Do the conclusions drawn in the research report flow from the analysis, or interpretation, of the data | Yes | Yes | Yes | Yes | Yes | Yes | Yes | Yes | Yes | Yes | Yes |
